# Neighborhood Disadvantage and Breast Cancer–Specific Survival

**DOI:** 10.1001/jamanetworkopen.2023.8908

**Published:** 2023-04-21

**Authors:** Neha Goel, Alexandra Hernandez, Cheyenne Thompson, Seraphina Choi, Ashly Westrick, Justin Stoler, Michael H. Antoni, Kristin Rojas, Susan Kesmodel, Maria E. Figueroa, Steve Cole, Nipun Merchant, Erin Kobetz

**Affiliations:** 1 Department of Surgery, Division of Surgical Oncology, University of Miami Miller School of Medicine, Miami, Florida; 2 Sylvester Comprehensive Cancer Center, University of Miami Miller School of Medicine, Miami, Florida; 3 Medical student, University of Miami Miller School of Medicine, Miami, Florida; 4 Department of Epidemiology, University of Michigan School of Public Health, Ann Arbor; 5 Department of Geography and Regional Studies, University of Miami Miller School of Medicine, Miami, Florida; 6 Department of Psychology, University of Miami Miller School of Medicine, Miami, Florida; 7 Department of Human Genetics, University of Miami Miller School of Medicine, Miami, Florida; 8 Department of Psychiatry/Biobehavioral Sciences and Medicine, University of California Los Angeles David Geffen School of Medicine, Los Angeles; 9 Department of Public Health Sciences, University of Miami Miller School of Medicine, Miami, Florida; 10 Division of Internal Medicine, Department of Medicine, University of Miami Miller School of Medicine, Miami, Florida

## Abstract

**Importance:**

Neighborhood-level disadvantage is an important factor in the creation and persistence of underresourced neighborhoods with an undue burden of disparate breast cancer–specific survival outcomes. Although studies have evaluated neighborhood-level disadvantage and breast cancer–specific survival after accounting for individual-level socioeconomic status (SES) in large national cancer databases, these studies are limited by age, socioeconomic, and racial and ethnic diversity.

**Objective:**

To investigate neighborhood SES (using a validated comprehensive composite measure) and breast cancer–specific survival in a majority-minority population.

**Design, Setting, and Participants:**

This retrospective multi-institutional cohort study included patients with stage I to IV breast cancer treated at a National Cancer Institute–designated cancer center and sister safety-net hospital from January 10, 2007, to September 9, 2016. Mean (SD) follow-up time was 60.3 (41.4) months. Data analysis was performed from March 2022 to March 2023.

**Exposures:**

Neighborhood SES was measured using the Area Deprivation Index (tertiles), a validated comprehensive composite measure of neighborhood SES.

**Main Outcomes and Measures:**

The primary outcome was breast cancer–specific survival. Random effects frailty models for breast cancer–specific survival were performed controlling for individual-level sociodemographic, comorbidity, breast cancer risk factor, access to care, tumor, and National Comprehensive Cancer Network guideline-concordant treatment characteristics. The Area Deprivation Index was calculated for each patient at the census block group level and categorized into tertiles (T1-T3).

**Results:**

A total of 5027 women with breast cancer were included: 55.8% were Hispanic, 17.5% were non-Hispanic Black, and 27.0% were non-Hispanic White. Mean (SD) age was 55.5 (11.7) years. Women living in the most disadvantaged neighborhoods (T3) had shorter breast cancer–specific survival compared with those living in the most advantaged neighborhoods (T1) after controlling for individual-level sociodemographic, comorbidity, breast cancer risk factor, access to care, tumor, and National Comprehensive Cancer Network guideline-concordant treatment characteristics (T3 vs T1: hazard ratio, 1.29; 95% CI, 1.01-1.65; *P* < .04).

**Conclusions and Relevance:**

In this cohort study, a shorter breast cancer–specific survival in women from disadvantaged neighborhoods compared with advantaged neighborhoods was identified, even after controlling for individual-level sociodemographic, comorbidity, breast cancer risk factor, access to care, tumor, and National Comprehensive Cancer Network guideline-concordant treatment characteristics. The findings suggest potential unaccounted mechanisms, including unmeasured social determinants of health and access to care measures. This study also lays the foundation for future research to evaluate whether social adversity from living in a disadvantaged neighborhood is associated with more aggressive tumor biologic factors, and ultimately shorter breast cancer-specific survival, through social genomic and/or epigenomic alterations.

## Key Points

**Question:**

Is living in a disadvantaged neighborhood associated with breast cancer–specific survival in a majority-minority population?

**Findings:**

In this cohort study of 5027 patients with breast cancer, neighborhood disadvantage was associated with shorter breast cancer–specific survival. This finding was noted after adjusting for individual-level sociodemographic, comorbidity, breast cancer risk factor, access to care, tumor, and National Comprehensive Cancer Network guideline-concordant treatment characteristics.

**Meaning:**

The findings of this study suggest unaccounted mechanisms associated with breast cancer–specific survival, such as unmeasured social and access to care barriers, and lays the foundation for future research evaluating whether neighborhood disadvantage leads to more aggressive tumor biologic factors through the accumulation of social and environmental stressors.

## Teaser

This cohort study examines the association of neighborhood-level disadvantage with breast cancer–specific survival in a racially and ethnically diverse population.

## Introduction

Advancements in screening, diagnosis, and treatment have led to a decrease in breast cancer rates across the US.^1,2,3,4^ However, not all local geographic areas (ie, neighborhoods) have benefited equally from these improved public health efforts, resulting in persistent breast cancer survival disparities.^5,6,7,8,9,10^ Neighborhood disadvantage remains a fundamental cause of health disparities in the US and contributes to the creation and persistence of underresourced neighborhoods with an undue burden of disparate health outcomes.^10,11,12,13^ Neighborhood disadvantage, therefore, warrants consideration as a significant ecologic risk factor when studying breast cancer survival inequities.

Studies have found associations between neighborhood-level measures, such as socioeconomic status (SES), with disparities in breast cancer survival.^14,15^ However, many measures of neighborhood SES previously used do not encapsulate the various domains and complexities that contribute to neighborhood disadvantage.^16^ Previous analyses also have methodologic limitations associated with confounding between neighborhood and individual-level factors or their design independently assesses only these measures of disadvantage rather than their joint outcome.^15,17^ Moreover, these studies predominantly use national cancer databases that have limited data on key variables, such as age (eg, only include individuals aged ≥65 years), SES (eg, do not include uninsured, Medicare-ineligible populations), and racial and ethnic diversity (eg, only 5% of patients are Hispanic) along with an inability to capture National Comprehensive Cancer Network (NCCN) guideline-concordant treatment.^18,19^

To overcome these data and methodologic limitations, we sought to assess whether there is an association between a robust validated measure of neighborhood disadvantage (Area Deprivation Index [ADI]) and breast cancer–specific survival in a diverse sociodemographic and racial and ethnic population with individual-level sociodemographic, comorbidity, breast cancer risk factor, access to care, tumor, and NCCN guideline-concordant treatment information not available in national cancer databases. In doing so, we intended to add to the literature by evaluating breast cancer–specific survival in women residing in disadvantaged neighborhoods, after controlling for detailed individual-level data to better isolate the outcomes associated with neighborhood disadvantage and breast cancer–specific survival, in a majority-minority (<50% non-Hispanic White)^20^ South Florida population, which has a complex history of residential segregation that has contributed to the formation of socially disadvantaged neighborhoods.^21^

## Methods

### Study Site and Population

**Figure 1.**
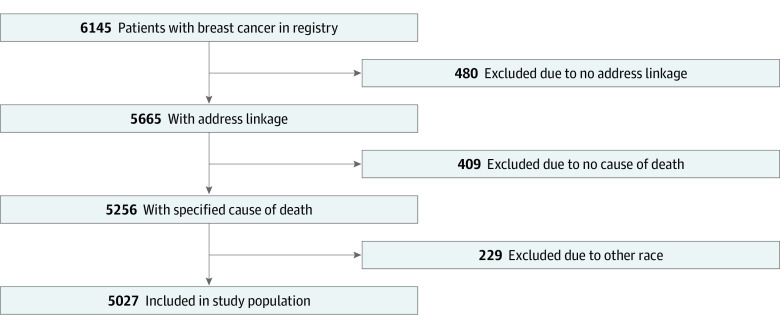
Study Flow Diagram

Institutional tumor registries were used to identify patients diagnosed and treated for stage I to IV breast cancer between January 10, 2007, to September 9, 2016, at a South Florida National Cancer Institute–designated cancer center and sister safety-net hospital. The catchment area includes Broward, Miami-Dade, Monroe, and Palm Beach counties. This region spans 10 000 square miles and is home to 6.2 million people, approximately 30% of Florida’s total population. Patients with ductal carcinoma in situ were excluded because this rarely affects breast cancer–specific survival.^22,23,24^ Patients with missing follow-up data were also excluded. Figure 1 details the study flow diagram. This cohort study was reviewed and approved by the institutional review board of the University of Miami. The need for informed consent was waived due to the use of deidentified data. We followed the Strengthening the Reporting of Observational Studies in Epidemiology (STROBE) reporting guideline.

### Covariates of Interest

Covariates included sociodemographic factors (age at diagnosis: <50, 50-69, 70-79, ≥80 years), race and ethnicity (Black Hispanic, White Hispanic [hereafter, Hispanic], non-Hispanic Black, and non-Hispanic White), birthplace (country other than US, US, and unknown), relationship status (divorced/separated, married, single, and unknown), comorbidities (body mass index [underweight, normal weight, overweight, and obese], diabetes [yes or no], hypertension [yes or no], coronary artery disease [yes or no]), breast cancer risk factors (tobacco use [never, active, former], alcohol use [never, active, former], age at menarche and menopause, current or history of exogenous hormone therapy [oral contraception, yes or no], postmenopausal hormone replacement therapy [yes or no], and family history of breast cancer [yes, no, or unknown]). Self-identified race and ethnicity was used as a sociopolitical construct and a proxy for structural racism. Insurance type was included as a measure of individual-level SES and access to care (private, Medicare, Medicaid, military, nonspecified insured, uninsured, and unknown).^25,26^ These data points are routinely collected on patient intake forms and were individually recorded from patient medical records. Tumor characteristics (clinical and pathologic stage [I, II, III, and IV]) and subtype (estrogen receptor [ER]-positive and *ERBB2-*negative [formerly *HER2* or *HER2/neu*], ER-positive/*ERBB2*-positive, ER-negative/*ERBB2-*negative, ER-negative/*ERBB2-*positive, and unknown), tumor grade (well or moderately, poorly, and anaplastic or undifferentiated) were collected from patient pathology reports. To account for treatment, adherence to NCCN stage and receptor-appropriate guidelines was determined by individual medical records review by 2 surgical oncologists (N.G. and S.K.) and treated as a dichotomous variable representing whether the patient completed or did not complete concordant treatment.^27^

### Primary Outcome

The primary outcome was breast cancer–specific survival, determined as time from primary diagnosis to point of death from any invasive local, regional, or distant event. Cause-specific death was determined by medical records review and treated as a dichotomous variable. Censoring was calculated using date of death (with cause of death) or last known follow-up.

### Area Deprivation Index

The ADI was used to measure neighborhood disadvantage. It is a validated, neighborhood-level composite index reflecting 17 dimensions of social determinants of health within the domains of housing, income, employment, and education, captured in the American Community Survey and US Census Survey data via principal components analysis methods. We used the 2015 ADI, which is a 5-year average of the American Community Survey data from 2011 to 2015. The ADI state rankings range from 1 to 10, with disadvantage reflected by higher scores.^28^ The state ADI composite score was calculated at the census block group level using the ADI mapping atlas and participant addresses.^28^

For analysis, state deciles were categorized into tertiles based on the literature.^29,30,31^ In addition, census block groups and study participants were most evenly distributed by tertiles in our cohort. Tertile 1 (T1) reflects the lowest ADI (most advantaged neighborhood) and T3 reflects the highest ADI (most disadvantaged neighborhood).

### Statistical Analysis

**Figure 2.**
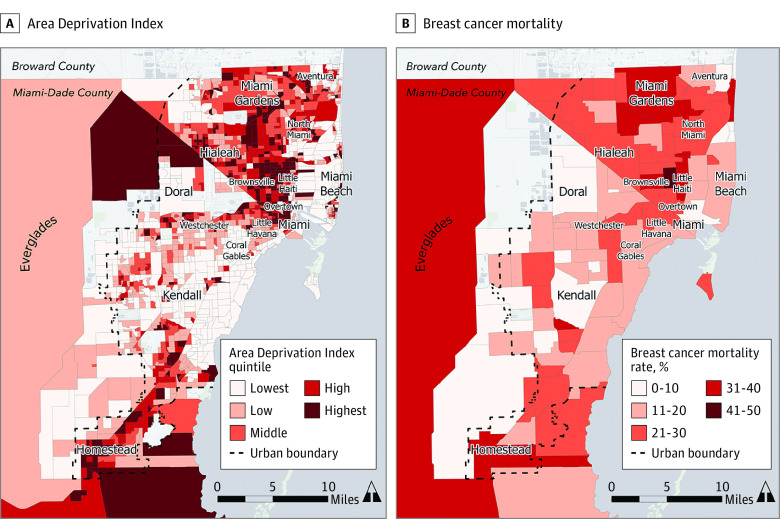
Geographic Patterns and Breast Cancer–Specific Mortality Neighborhood Area Deprivation Index (A) and breast cancer–specific mortality rates (B) in Miami-Dade County.

Data analysis was performed from March 2022 to March 2023. Descriptive statistics with χ^2^ and analysis of variance for categorical variables and *t* tests for continuous variables were conducted by ADI tertiles. A multilevel analysis was conducted to account for the hierarchical nature of patients nested within census block groups. Previous studies have shown that cancer rates are similar at census tract and block group levels with minimal bias due to unstable rates.^32,33^ To evaluate the association between ADI tertiles and breast cancer–specific survival, univariate and multilevel Cox proportional hazards regression model analysis was conducted, controlling for age, race and ethnicity, insurance, receptor status, body mass index, hypertension, diabetes, and NCCN guideline-concordant treatment. Kaplan-Meier survival curves were calculated by ADI tertiles for breast cancer–specific survival. We identified these covariates to include based on the literature, subject matter knowledge, and to optimize model fit.^2,21,34,35,36,37,38^ We then used census block group-level ADI values for Miami-Dade County to qualitatively assess geographic patterns and breast cancer–specific mortality at the neighborhood level (an aggregation of block groups) (Figure 2). All analyses were conducted using R, version 3.5.2, using survival version 2.38, survminer version 0.4.3, and coxme version 2.2-10 (R Foundation for Statistical Computing). All statistical tests were 2-sided, and statistical significance was assessed at α < .05.

## Results

### Sociodemographic, Comorbidities, Breast Cancer Risk Factors, and Access to Care Characteristics by ADI Tertiles

The study comprised 5027 patients with breast cancer. Most of the population was Hispanic (55.8%), 1371 (27.3%) were non-Hispanic White, and 853 (17.0%) were non-Hispanic Black. The mean (SD) age was 55.5 (11.7) years. Non-Hispanic Black patients were more likely to live in neighborhoods with the highest area disadvantage compared with non-Hispanic White patients (28.6% vs 13.3%; *P* < .001). Single patients compared with married patients were more likely to live in neighborhoods with higher disadvantage (41.7% vs 36.9%; *P* < .001). Patients with Medicaid insurance were more likely to live in areas with the highest neighborhood disadvantage (ADI T3) than patients in the lowest ADI tertile (27.4% vs 9.7%; *P* < .001). Patients in the highest ADI tertile were also more likely to be uninsured (21.3% vs 9.2%; *P* < .001). Patients living in the most disadvantaged neighborhoods were more likely to be obese (41.5% vs 22.5%; *P* < .001) and have diabetes (9.6% vs 4.7%; *P* < .001) compared with those living in areas with the lowest disadvantage (Table 1). The number of census block groups included in ADI T1 was 21; in T2, 18; and in T3, 27.

**Table 1.** Patient Sociodemographic and Breast Cancer Risk Factors by Area Deprivation Index Tertile

Factor	**No. (%)**				*P* value
	Tertile 1 (n = 1963)	Tertile 2 (n = 1488)	Tertile 3 (n = 1576)	Total (N = 5027)	
Block groups	21	18	27	66	
Sociodemographic					
Age, mean (SD)	55.75 (11.97)	55.15 (11.35)	55.57 (11.77)	55.52 (11.73)	.32
Age, y					
<50	636 (32.4)	455 (30.6)	479 (30.4)	1570 (31.2)	.90
50-69	1065 (54.3)	876 (58.9)	904 (57.4)	2845 (56.6)	
70-79	195 (9.9)	119 (8.0)	150 (9.5)	464 (9.2)	
≥80	67 (3.4)	38 (2.6)	43 (2.7)	148 (2.9)	
Race and ethnicity					
Hispanic Black	15 (0.8)	28 (1.9)	47 (3.0)	90 (1.8)	<.001
Hispanic White	938 (47.8)	906 (60.9)	869 (55.1)	2713 (54.0)	
Non-Hispanic Black	123 (6.3)	279 (18.8)	451 (28.6)	853 (17.0)	
Non-Hispanic White	887 (45.2)	275 (18.5)	209 (13.3)	1371 (27.3)	
Birthplace					<.001
US born	822 (41.9)	452 (30.4)	531 (33.7)	1805 (35.9)	
Other country	583 (29.7)	781 (52.5)	799 (50.7)	2163 (43.0)	
Unknown	558 (28.4)	255 (17.1)	246 (15.6)	1059 (21.1)	
Relationship status					
Divorced/separated	243 (12.4)	277 (18.6)	295 (18.7)	815 (16.2)	<.001
Married	1178 (60.0)	640 (43.0)	582 (36.9)	2400 (47.7)	
Single	497 (25.3)	534 (35.9)	657 (41.7)	1688 (33.6)	
Unknown	45 (2.3)	37 (2.5)	42 (2.7)	124 (2.5)	
Comorbidities and breast cancer risk factors					
BMI					
Underweight (<18.5)	19 (1.1)	11 (0.9)	15 (1.1)	45 (1.0)	<.001
Normal weight (18.5-24.9)	653 (38.8)	320 (24.8)	316 (22.8)	1289 (29.5)	
Overweight (25.0-29.9)	560 (33.3)	478 (37.1)	482 (34.7)	1520 (34.8)	
Obese (>29.9)	452 (26.8)	481 (37.3)	576 (41.5)	1509 (34.6)	
Diabetes	93 (4.7)	110 (7.4)	151 (9.6)	354 (7.0)	<.001
Hypertension	441 (22.5)	404 (27.2)	422 (26.8)	1267 (25.2)	.002
Coronary artery disease	9 (0.5)	5 (0.3)	9 (0.6)	23 (0.5)	.63
Tobacco use					
Never	1220 (67.7)	990 (70.7)	1059 (70.3)	3269 (69.4)	<.001
Active	91 (5.1)	137 (9.8)	147 (9.8)	375 (8.0)	
Former	490 (27.2)	273 (19.5)	300 (19.9)	1063 (22.6)	
Alcohol use					
Never	1008 (56.1)	1087 (77.8)	1211 (80.7)	3306 (70.4)	<.001
Active	780 (43.4)	296 (21.2)	278 (18.5)	1354 (28.8)	
Former	8 (0.4)	14 (1.0)	12 (0.8)	34 (0.7)	
Age at menarche, mean (SD), y	12.59 (1.68)	12.63 (1.69)	12.67 (1.87)	12.63 (1.75)	.55
Age at menopause, mean (SD), y	47.70 (5.85)	47.52 (6.08)	47.02 (6.72)	47.42 (6.22)	.04
Current or history of exogenous hormone therapy					
Oral contraception	587 (50.5)	299 (35.4)	273 (31.3)	1159 (40.3)	<.001
Postmenopausal HRT	250 (22.7)	100 (12.3)	98 (11.7)	448 (16.3)	<.001
Family history of breast cancer	732 (42.6)	475 (35.1)	503 (34.2)	1710 (37.6)	<.001
Access to care					
Insurance					
Private	1169 (59.6)	575 (38.6)	490 (31.1)	2234 (44.4)	<.001
Medicaid	191 (9.7)	361 (24.3)	432 (27.4)	984 (19.6)	
Medicare	170 (8.7)	107 (7.2)	138 (8.8)	415 (8.3)	
Military	16 (0.8)	8 (0.5)	17 (1.1)	41 (0.8)	
Insured, NOS	101 (5.1)	92 (6.2)	80 (5.1)	273 (5.4)	
Uninsured	181 (9.2)	282 (19.0)	336 (21.3)	799 (15.9)	
Unknown	135 (6.9)	63 (4.2)	83 (5.3)	281 (5.6)	

Abbreviations: BMI, body mass index (calculated as weight in kilograms divided by height in meters squared); HRT, hormone replacement therapy; NOS, not otherwise specified.

### Tumor Characteristics and Receipt of NCCN Guideline-Concordant Treatment by ADI Tertiles

Patients living in the most disadvantaged neighborhoods (T3) compared with the most advantaged neighborhoods (T1) were more likely to have triple-negative breast cancer (17.4% vs 13.3%; *P* = .001), poorly differentiated tumors (42.1% vs 37.8%; *P* = .001), higher-stage disease at presentation (stage III, 20.4%, and stage IV, 11.2% vs stage III, 14.3%, and stage IV, 7.1%; *P* < .001), and were less likely to complete NCCN guideline-concordant treatment (75.4% vs 82.1%; *P* < .001) compared with the most advantaged neighborhoods (Table 2).

**Table 2.** Tumor Characteristics and Receipt of NCCN Guideline-Concordant Treatment by Area Deprivation Index

Factor	Tertile 1 (n = 1963)	Tertile 2 (n = 1488)	Tertile 3 (n = 1576)	Total (n = 5027)	*P* value
Receptor status					
ER-positive/*ERBB2-*negative	1226 (62.5)	897 (60.3)	928 (58.9)	3051 (60.7)	.001
ER-positive/*ERBB2-*positive	226 (11.5)	161 (10.8)	168 (10.7)	555 (11.0)	
ER-negative/*ERBB2-*negative	262 (13.3)	239 (16.1)	274 (17.4)	775 (15.4)	
ER-negative/*ERBB2-*positive	120 (6.1)	116 (7.8)	134 (8.5)	370 (7.4)	
Unknown	129 (6.6)	75 (5.0)	72 (4.6)	276 (5.5)	
Clinical stage					
I	864 (44.0)	552 (37.1)	519 (32.9)	1935 (38.5)	<.001
II	680 (34.6)	555 (37.3)	560 (35.5)	1795 (35.7)	
III	280 (14.3)	254 (17.1)	321 (20.4)	855 (17.0)	
IV	139 (7.1)	127 (8.5)	176 (11.2)	442 (8.8)	
Tumor grade					
Well/moderately differentiated	1216 (61.9)	871 (58.5)	898 (57.0)	2985 (59.4)	.003
Poorly differentiated	742 (37.8)	603 (40.5)	664 (42.1)	2009 (40.0)	
Anaplastic/undifferentiated	5 (0.3)	14 (0.9)	14 (0.9)	33 (0.7)	
Final pathologic stage					
0	15 (0.8)	8 (0.5)	9 (0.6)	32 (0.6)	<.001
I	859 (43.8)	523 (35.1)	497 (31.5)	1879 (37.4)	
II	519 (26.4)	419 (28.2)	375 (23.8)	1313 (26.1)	
III	172 (8.8)	146 (9.8)	166 (10.5)	484 (9.6)	
IV	50 (2.5)	44 (3.0)	50 (3.2)	144 (2.9)	
Unknown	346 (17.6)	347 (23.3)	477 (30.3)	1170 (23.3)	
Treatment					
Surgery	1706 (86.9)	1186 (79.7)	1153 (73.2)	4045 (80.5)	<.001
Chemotherapy	1098 (55.9)	866 (58.2)	896 (56.9)	2860 (56.9)	.511
Radiotherapy	987 (50.3)	697 (46.8)	690 (43.8)	2374 (47.2)	<.001
Endocrine therapy	1248 (63.6)	827 (55.6)	814 (51.6)	2889 (57.5)	<.001
NCCN guideline-Concordant Treatment	1611 (82.1)	1142 (76.7)	1188 (75.4)	3941 (78.4)	<.001

Abbreviations: ER, estrogen receptor; NCCN, National Comprehensive Cancer Network.

### Breast Cancer–Specific Survival by ADI Tertile

The mean (SD) follow-up time for the study participants was 60.3 (41.4) months overall. The mean (SD) follow-up time for patients who were alive was 64.2 (41.6) months and 34.9 (29.8) months for patients who died. Follow-up times by ADI tertiles were similar across groups: T1, 60.0 (41.6) months; T2, 61.7 (41.4) months; and T3, 59.3 (41.2) months. On univariate analysis for breast cancer–specific survival, we found that the disadvantaged neighborhoods (T2 and T3) had a higher risk of breast cancer mortality (T2: hazard ratio [HR], 1.36; 95% CI, 1.11-1.66; T3: HR, 1.77; 95% CI, 1.46-2.15). Kaplan-Meier survival analysis curves by ADI tertiles also showed a significant difference between groups (*P*<.001) (eFigure 1 in Supplement 1). On multilevel Cox proportional hazards modeling for breast cancer–specific survival, we found that patients living in the most disadvantaged neighborhoods (T3) had higher HRs of breast cancer–specific mortality compared with those living in the most advantaged neighborhoods (T1), after controlling for individual-level factors, tumor characteristics, and NCCN-guideline appropriate treatment (T3 HR, 1.44; 95% CI, 1.13-1.84; *P* = .003). Additional factors associated with increased breast cancer–specific mortality were non-Hispanic Black race (HR, 1.70; 95% CI, 1.26-2.30; *P* < .001) and aggressive tumor subtype (ER-negative/*ERBB2-*negative) (HR, 2.07; 95% CI, 1.67-2.56; *P* < .001) (Table 3). As shown in Figure 2, we observed similar geographic distributions between neighborhoods with increased breast cancer mortality and areas with the highest ADI.

**Table 3.** Multivariable Frailty Model for Hazards of Breast Cancer–Specific Mortality

Variable	HR (95% CI)	*P* value
Area Deprivation Index		
Tertile 1 (most advantaged)	1 [Reference]	
Tertile 2	1.22 (0.96-1.56)	.10
Tertile 3	1.44 (1.13-1.84)	.003
Age	1.02 (1.01-1.02)	<.001
Race and ethnicity		
Hispanic^a^	0.94 (0.72-1.23)	.64
Non-Hispanic Black	1.70 (1.26-2.30)	<.001
Non-Hispanic White	1 [Reference]	
Insurance		
Private	1 [Reference]	
Medicaid	0.96 (0.62-1.48)	.83
Medicare	1.44 (1.13-1.84)	.002
Insurance, NOS	1.34 (0.95-1.89)	.09
Uninsured	1.12 (0.84-1.49)	.43
Unknown	1.14 (0.74-1.76)	.55
Receptor status		
ER-positive/*ERBB2-*negative	1 [Reference]	
ER-positive/*ERBB2-*positive	1.38 (1.04-1.83)	.02
ER-negative/*ERBB2-*negative	2.07 (1.67-2.56)	<.001
ER-negative/*ERBB2-*positive	1.18 (0.84-1.67)	.33
Unknown	0.93 (0.54-1.59)	.78
BMI		
Normal (18.5-24.9)	1 [Reference]	
Underweight (<18.5)	1.41 (0.73-2.73)	.29
Overweight (25.0-29.9)	0.68 (0.55-0.86)	<.001
Obese (>29.9)	0.77 (0.62-0.96)	.02
Hypertension	0.85 (0.69-1.05)	.13
Diabetes	1.05 (0.76-1.44)	.76
Receipt of NCCN guideline-concordant treatment	0.85 (0.76-0.95)	.003

Abbreviations: BMI, body mass index (calculated as weight in kilograms divided by height in meters squared); HR, hazard ratio; NCCN, National Comprehensive Cancer Network; NOS, not otherwise specified.

^a^ Hispanic Black and Hispanic White patients were not evaluated separately because the number of participants in the Hispanic Black subgroup was very low.

## Discussion

This study found that neighborhood disadvantage independently associated with shorter breast cancer–specific survival in a socioeconomically, racially and ethnically, and age-diverse majority-minority population. These disparities remained even after accounting for individual-level sociodemographic, comorbidity, breast cancer risk factor, access to care, tumor, and NCCN guideline-concordant treatment characteristics, not available in national database studies,^18,19^ suggesting unaccounted mechanisms through which neighborhood disadvantage may be associated with shorter breast cancer–specific survival.

Our study expands on the literature in many important ways. To our knowledge, this is the first study to evaluate neighborhood disadvantage and breast cancer–specific survival in a majority-minority population, thus expanding generalizability and addressing important goals of increasing diversity in high-impact scientific journals.^39,40,41^ National databases, such as Surveillance, Epidemiology, and End Results Program and National Cancer Database, are well known to have an underrepresentation of racial and ethnic minority populations, which limits studies that use these databases.^18,19,42^ Even regional databases that may have larger representations of non-Hispanic Black patients lack ethnic diversity and usually have very small Hispanic populations.^36,43^ Our study population is among the most diverse in the nation in terms of race and ethnicity, ancestry, and cultural identity with nearly half of South Florida residents born in Latin America or the Caribbean.^35^ Moreover, our analysis controls for race and ethnicity as a proxy for structural racism. This strengthens the argument that neighborhoods themselves, larger structures that promote inequities for racial and ethnic minority populations, are also associated with disparities across all races and ethnicities. By taking the focus away from individual-level race and ethnicity, we add novel insight to the literature to dismantle racialized-biological differences and age-old racist beliefs as the only cause of breast cancer–specific survival.^44,45^

Our findings also improve generalizability beyond just evaluation of a majority-minority population. Large national databases are limited in their exclusion of non-Medicare populations to evaluate more diverse populations. A study by Cheng et al^7^ importantly found associations between ADI and breast cancer survival while accounting for individual-level SES, tumor, and treatment; however, this study was limited to patients receiving Medicare, which limits generalizability. By using institutional registry data, we were able to capture women younger than 65 years, which, to our knowledge, has not been previously analyzed through the lens of neighborhood-level disadvantage after controlling for individual-level risk factor, sociodemographic, access to care, tumor, and NCCN guideline-concordant treatment factors. Specifically, by controlling for non-Medicare insurance types (uninsured, private, Medicaid, military, nonspecified insured), we were able to generalize the neighborhood disadvantage along the uninsured-insured spectrum.

Moreover, our study controlled for granular individual-level comorbidity and risk factor data not available in regional or national databases, such as obesity and diabetes. These are known risk factors for breast cancer, particularly triple-negative breast cancer, a more aggressive subtype associated with worse survival outcomes, which is also more commonly seen in disadvantage neighborhoods.^46,47,48,49^ In addition, we controlled for NCCN guideline-concordant treatment, a variable that is unavailable in both regional and large national databases.^7,43^ By controlling for these additional confounders that, to our knowledge, have not been examined in other studies, we expand on previous literature to further isolate the association between neighborhood disadvantage and breast cancer–specific survival.^42,50^

Combined, this persistent disparity associated with neighborhood disadvantage on breast cancer–specific survival disparities, even after accounting for individual-level sociodemographic, tumor, and treatment characteristics, suggests unaccounted mechanisms. These unmeasured inequities exist along the breast cancer care continuum from delays in diagnosis to lack of treatment completion. For example, in disadvantaged neighborhoods, lack of access to health care resources and appropriate referrals can delay diagnosis and lead to later stage at presentation, which can result in shorter breast cancer–specific survival.^14,51,52^ Lack of transportation and poor social support may affect the completion of appropriate treatment, which is associated with shorter breast cancer–specific survival.^8,53^ In addition to these unmeasured social barriers, our findings raise the question as to whether neighborhood disadvantage leads to more aggressive tumor biological factors and ultimately shorter breast cancer–specific survival.

The field of social genomics has established that stress and social adversity lead to stress-related genomic alterations that adversely affect tumor biology.^34,54,55,56,57^ Specifically, studies have identified that repeated psychological stressors, such as social isolation, place demands on the hypothalamic-pituitary-adrenal axis and sympathetic nervous system, upregulating proinflammatory signaling, which leads to more aggressive tumor biological factors.^58,59,60^ These stress-related neuroendocrine signaling pathways of the sympathetic nervous system lead to a pattern of social adversity-associated blood leukocyte gene expression, termed the *conserved transcriptional response to adversity* (CTRA).^54,60,61,62^ However, whether the stress associated with living in a disadvantaged neighborhood (eg, due to increased violence or poverty) also upregulates blood leukocyte CTRA gene and tumor gene expression of proinflammatory pathways associated with shorter breast cancer–specific survival has yet to be established. The conceptual model to operationalize our findings and further investigate these associations can be seen in eFigure 2 in Supplement 1. In addition to social genomics, the field of social epigenomics also translates social and environmental adversity into consequential biological changes associated with stress and inflammation.^13,63,64,65,66^ Studies have demonstrated a link between global and gene-specific DNA methylation patterns associated with socially patterned stressors, including low adult SES,^66,67^ perceived neighborhood stress,^64^ and neighborhood crime.^68^ A study of 1226 participants of the Multi-Ethnic Study of Atherosclerosis, a population-based sample of US adults, found that neighborhood socioeconomic disadvantage and the social environment are associated with differential DNA methylation of proinflammatory genes (eg, *F8*, *TLR1*).^13^ These findings remain to be validated in breast cancer.

### Limitations

This study has limitations. Along with inherent limitations of retrospective studies, we were unable to capture potential treatments received at other facilities in approximately 4% of the cases. In addition, although we captured hypertension, diabetes, and coronary artery disease, we were unable to capture Charlson Comorbidity Index levels, which may affect completion of treatment or lead to chemotherapy dose reductions, ultimately also affecting survival outcomes. Another limitation is that individual-level insurance coverage was utilized as a proxy for access to care, which does not comprehensively represent all access to care measures. Moreover, this was a this was a 2-institution study, which limits generalizability. Nevertheless, our catchment area included Broward, Miami-Dade, Monroe, and Palm Beach counties, an area that spans 10 000 square miles and is home to 6.2 million people, approximately 30% of Florida’s total population. Moreover, the use of ADI allowed us to analyze neighborhood disadvantage in a more comprehensive and nuanced way than earlier studies.^3,14^ Studies by Shariff-Marco et al^69^ and Banegas et al^70^ were some of the first to highlight the importance of assessing both individual and neighborhood SES and created their own composite neighborhood-level SES scores for their analyses. The ADI importantly expands on past scores to include more domains of neighborhood deprivation, is widely validated, and is a more detailed score with a smaller geographic unit (ie, census block group) than other widely used measures of neighborhood disadvantage, such as the Yost Index.^71^ Although our neighborhood characteristics are drawn from publicly available data sets and not perfectly temporally synchronized to patients' year of diagnosis, they still serve as a proxy for block-group-level disparities.^7^ In addition, although we studied state-level ADI in our final model, we found that national-level ADI measures for the patients were similar to our state-level ADI values, further suggesting our findings are generalizable.^7^ Another strength of this study lies in its ability to capture granular data on individual-level characteristics, tumor characteristics, such as *ERBB2* status, and receipt of NCCN guideline-concordant treatment, which are not available or accounted for in earlier national database studies.^7,72^ Having this level of detailed information allows us to hypothesize that persistent disparities in breast cancer–specific survival by neighborhood disadvantage might be associated with underlying biological mechanisms leading to aggressive tumor biological factors among those living in disadvantaged neighborhoods compared with advantaged neighborhoods. Nevertheless, in addition to these aforementioned biologic mechanisms, other nonbiologic pathways that are not accounted for in our analysis may also be contributing to these residual disparities in breast cancer–specific survival.^21,35,44,73,74^

## Conclusions

In this retrospective cohort study, we identified neighborhood disadvantage as an independent factor associated with shorter breast cancer–specific survival in a socioeconomically, racially and ethnically, and age-diverse majority-minority population. Our study findings suggest unaccounted mechanisms associated with shorter breast cancer–specific survival among women from disadvantaged neighborhoods even after accounting for established multilevel factors associated with shorter breast cancer–specific survival, particularly those associated with access to care. One hypothesis that merits further inquiry is more aggressive tumor biologic factors among women from disadvantaged compared with advantaged neighborhoods. This study therefore may advance the field of breast cancer disparities research by suggesting additional pathways by which neighborhood disadvantage may affect breast cancer–specific survival disparities beyond access to care. This strengthens the call to action for future research on the biologic mechanisms by which neighborhood disadvantage affects breast cancer biologic factors and ultimately breast cancer–specific survival.

## Notes

Supplement 1. **eFigure 1.** Kaplan-Meier Survival Curves for Breast Cancer–Specific Survival by Area Deprivation Index Tertiles **eFigure 2.** Conceptual Model for the Effects of Neighborhood Disadvantage on Tumor Biology

Supplement 2.
Data Sharing Statement
