# Supplementary material for: Neighborhood Disadvantage and Breast Cancer–Specific Survival
Source: JAMA Netw Open. 2023 Apr 21;6(4):e238908. doi: 10.1001/jamanetworkopen.2023.8908 (PMC10122178; doi:10.1001/jamanetworkopen.2023.8908)
Supplement: Supplement 1. — eFigure 1. Kaplan-Meier Survival Curves for Breast Cancer–Specific Survival by Area Deprivation Index Tertiles eFigure 2. Conceptual Model for the Effects of Neighborhood Disadvantage on Tumor Biology [file jamanetwopen-e238908-s001.pdf]

Supplementary Online Content

Goel N, Hernandez A, Thompson C, et al. Neighborhood disadvantage and breast cancer–specific survival. *JAMA Netw Open*. 2023;6(4):e238908. doi:10.1001/jamanetworkopen.2023.8908

eFigure 1. Kaplan-Meier Survival Curves for Breast Cancer–Specific Survival by Area Deprivation Index Tertiles

eFigure 2. Conceptual Model for the Effects of Neighborhood Disadvantage on Tumor Biology

This supplementary material has been provided by the authors to give readers additional information about their work.

© 2023 Goel N et al. *JAMA Network Open.*

eFigure 1. Kaplan-Meier Survival Curves for Breast Cancer–Specific Survival by Area Deprivation Index Tertiles.

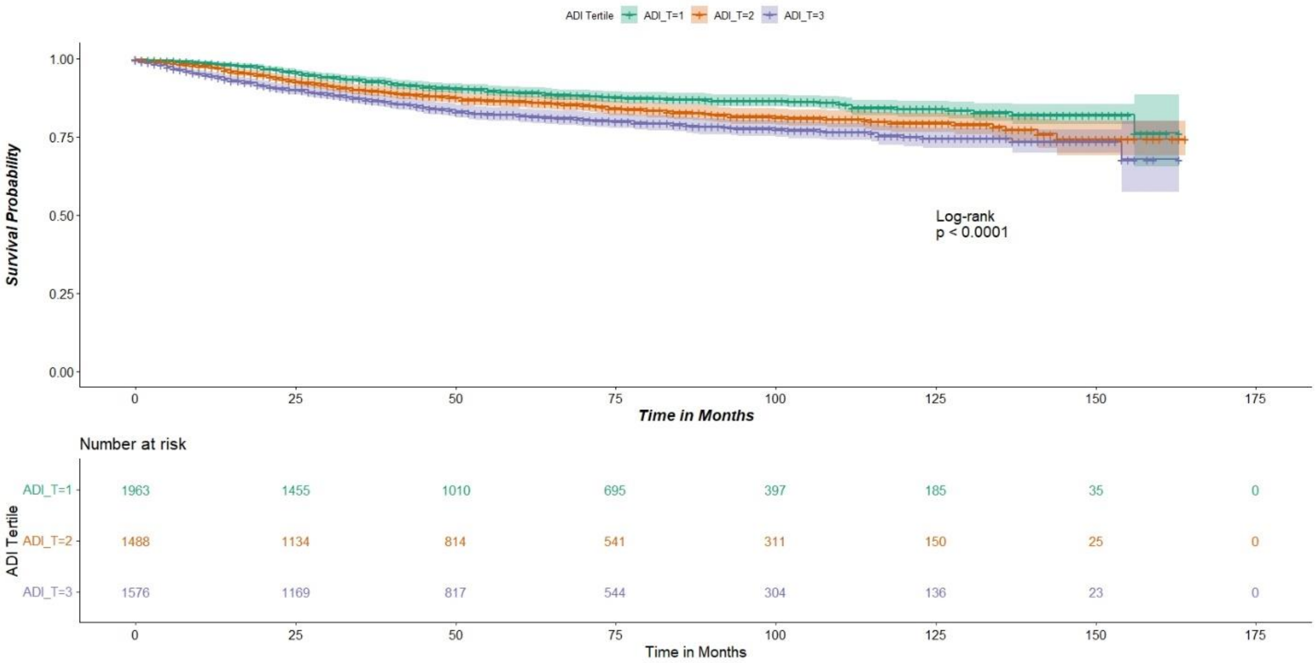

© 2023 Goel N et al. *JAMA Network Open.*

eFigure 2. Conceptual Model for the Effects of Neighborhood Disadvantage on Tumor Biology. This conceptual model illustrates how objective and subjective social adversity activate the sympathetic nervous system (SNS) leading to upregulation of pro-inflammatory pathways and more aggressive tumor biology.

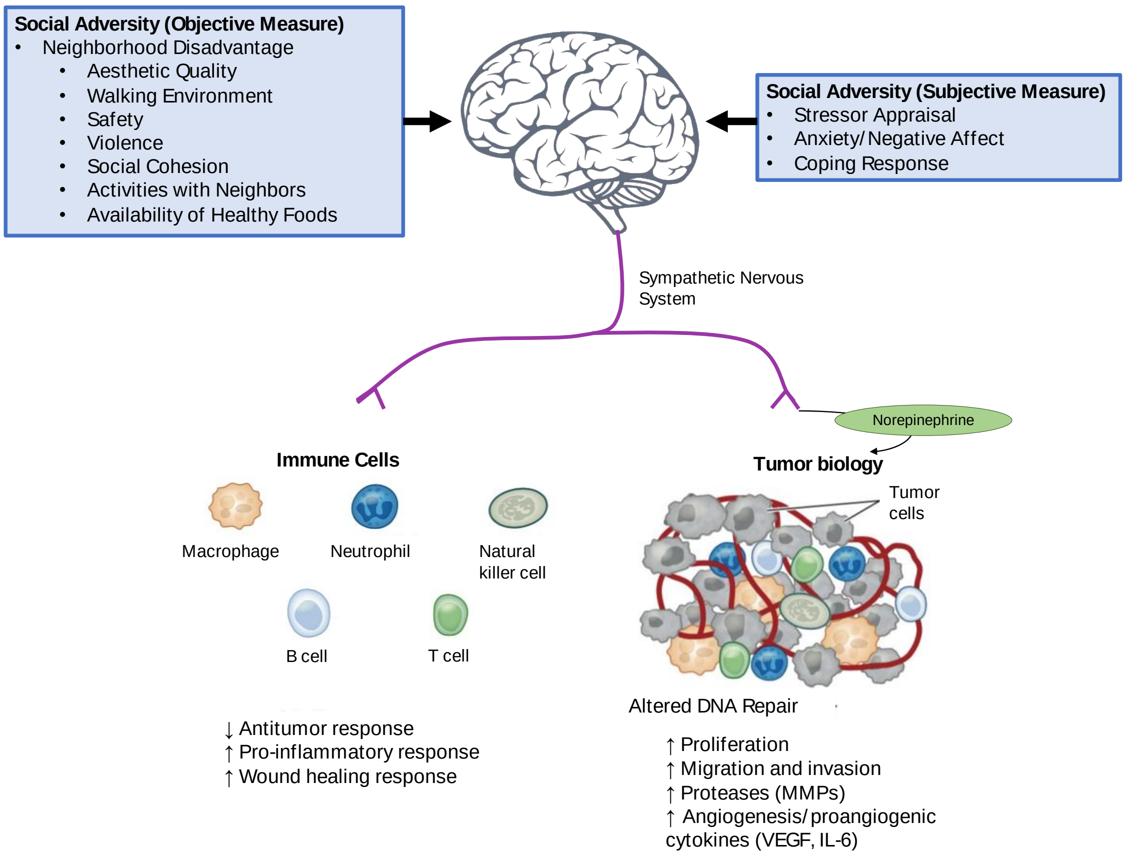

© 2023 Goel N et al. *JAMA Network Open.*
