# Supplementary material for: Neighborhood Disadvantage and Breast Cancer–Specific Survival
Source: JAMA Netw Open. 2023 Apr 21;6(4):e238908. doi: 10.1001/jamanetworkopen.2023.8908 (PMC10122178; doi:10.1001/jamanetworkopen.2023.8908)
Supplement: Supplement 2. — Data Sharing Statement [file jamanetwopen-e238908-s002.pdf]

Data Sharing Statement

Goel. Neighborhood Disadvantage and Breast Cancer-Specific Survival. *JAMA Netw Open*. Published April 21, 2023. doi:10.1001/jamanetworkopen.2023.8908

Data
Data available: Yes
Data types: Deidentified participant data
How to access data: The author Dr. Neha Goel can be reached at neha.goel@med.miami.edu and will make data available upon reasonable request.
When available: With publication

Supporting Documents
Document types: None

Additional Information
Who can access the data: researchers whose proposed use of the data has been approved
Types of analyses: specified pre-approved purpose
Mechanisms of data availability: after approval of a proposal
